# Biodegradable Food Packaging Materials and Prospects of the Fourth Industrial Revolution for Tomato Fruit and Product Handling

**DOI:** 10.1155/2020/8879101

**Published:** 2020-11-21

**Authors:** S. M. Chisenga, G. N. Tolesa, T. S. Workneh

**Affiliations:** ^1^School of Engineering, Bioresources Engineering, University of KwaZulu-Natal, Pietermaritzburg, South Africa; ^2^Department of Food Science and Postharvest Technology, Haramaya Institute of Technology, Haramaya University, Dire Dawa, Ethiopia

## Abstract

The environment and food safety are major areas of concern influencing the development of biodegradable packaging for partial replacement of petrochemical-based polymers. This review is aimed at updating the recent advances in biodegradable packaging material and the role of virtual technology and nanotechnology in the tomato supply chain. Some of the common biodegradable materials are gelatin, starch, chitosan, cellulose, and polylactic acid. The tensile strength, tear resistance, permeability, degradability, and solubility are some of the properties defining the selection and utilization of food packaging materials. Biodegradable films can be degraded in soil by microbial enzymatic actions and bioassimilation. Nanoparticles are incorporated into blended films to improve the performance of packaging materials. The prospects of the fourth industrial revolution can be realized with the use of virtual platforms such as sensor systems in authentification and traceability of food and packaging products. There is a research gap on the development of a hybrid sensor system unit that can integrate sampling headspace (SHS), detection unit, and data processing of big data for heterogeneous tomato-derived volatiles. Principal component analysis (PCA), linear discriminant analysis (LDA), and artificial neutral network (ANN) are some of the common mathematical models for data interpretation of sensor systems.

## 1. Introduction

The global population is about 7.8 billion in 2020 and is estimated to reach 10 billion in 2050 [1]. The increasing population, urbanization, variability in diet, and climate change put pressure on food security including postharvest of fresh produce. The major loss of fresh produce occurs at the postharvest stage [2]. The fresh produce including tomato fruit is perishable due to high moisture content [2]. Generally, postharvest losses (30-50%) of the fresh produce are associated with handling, storage, and packaging. The bulk nature of produce along the supply chain makes it difficult to monitor and control losses. Nevertheless, digital technologies including smart packaging innovations are considered suitable for tracking and controlling postharvest losses. The application of these smart logistic technologies finds use in product traceability systems on information that relates the product to its genetic factors and environmental conditions [3]. Furthermore, IFPRI [3] stressed that digitalization focused on the ecosystem including agricultural production, processing, transportation, and market system can enhance the food value chain and improve competitiveness. This necessitated the concept of digitalization of logistic systems including food packaging and market services. However, the use of synthetic plastic materials in food packaging can have an adverse effect on climate and the environment [4]. Hence, eco-friendly packaging materials are increasingly becoming the alternative. Muller et al. [4] reported that polylactic acid and starch are potential materials to replace synthetic polymer films, i.e., plastics food packaging materials. Moreover, Jeevahan et al. [5] reported that edible biofilms are compostable and can be manufactured from the polysaccharides, proteins, and lipids. The production of edible biofilms is a recent approach to generate biodegradable food packaging safe to humans and the environment. In addition, Guerrini et al. [6] reported that biodegradable films have physicochemical and mechanical properties suitable to replace common polymerplastic applications. However, the food industries are facing a range of challenges from climate change, increasing consumer safety demands, and subsequent issues relating to government policies and legislative requirements [7]. The environmental concerns associated with the nonbiodegradable nature of plastic biopolymers are impacting negatively on the ecosystem. In view of this, there is an increasing demand to replace synthetic plastic materials with biodegradable materials. This review is aimed at gathering recent advances in biodegradable packaging film materials and their performance on the quality of tomato. The role of virtual technology and nanotechnology in the tomato supply chain is highlighted in response to fourth industrial revolution.

## 2. Importance of Food Packaging

The economic value of packaging is reflected in the packaging conversion industry, packaging supply chain, and in the retail industry. In 2015, the packaging industry recorded revenues of $839 billion worldwide [8] and was projected to grow by 3.5% by 2020. Western Europe and Americas are the largest consumers of packaging. The packaging industry contributes ~2% to gross domestic product (GDP) of the South African economy. The global demand for bio-based food packaging material is forecasted to reach ~1 million tons per year by 2020 [9]. The packaging material is considered as the major component in the sustainable development goal number 12 focused on themes (climate action, ocean action, plastic pollution in the ocean, food loss and waste, and sustainable transport) that relate to sustainable consumption and production. Food packaging provides protection and preservation of food by making a physical barrier against contamination due to foreign matter and environmental-related factors. This ultimately contributes to extended shelf life of food product. Other functions include mechanical and physical strength, convenience, and communication through product labeling [10, 11]. The actors in the value chain specific to food packaging include food processors, farmers, retailers, and researchers [12]. Postharvest strategy of minimizing loss through the packaging of tomato along the supply chain results in extended shelf life, improved income, livelihood, and food security [13]. Recent development in novel food packaging is driven by consumer's demand for convenience, ready to eat food, shelf stability, and maintenance of food quality [14]. The plastic polymers have been utilized for food packaging material production due to their availability and simplicity of manufacturing [15]. The petroleum polymers are hardly degradable and thus causing defects in the ecosystem [15]. Moreover, O'Brine and Thompson [16] reported that polymer plastic materials may take over 100 years to decompose. Similarly, Webb et al. [17] reported that polymer plastics that are landfilled could take longer than 20 years with no change in the plastic property. Hence, there are developments to replace petroleum-derived plastic with biodegradable materials. The innovation and development of food packaging from renewable, compostable, and biodegradable to active and intelligent packaging were reported [18, 19]. In addition, barrier properties, compatibility materials, and shelf life extension properties of the innovative packaging determine selection and utilization [18, 19]. The environmental safety concerns are limiting the use of plastic films for packaging in the food industries. Consequently, biopolymer films are receiving attention due to their biodegradable properties.

## 3. Overview of Biodegradable Packaging

The utilization of biodegradable materials on the markets of North America, Europe, and Asia has grown in the range of 15-20% CAGR from 2012 to 2017 [20] but the market data for Africa is not well established. Atarés and Chiralt [21] reported the application of essential oil in biodegradable food packaging films in Spain for the production of bio-based packages with potential health benefits (antioxidants and antimicrobial properties). The lipid nature of essential oils can decrease water vapor permeability in hydrophilic materials and can also improve the structural, mechanical, and optical properties of packaging films. The biodegradable packaging films developed and tested on tomato fruit in Finland for preservation objectives resulted in extended shelf life [22]. In Malaysia, Ali et al. [23] demonstrated the use of gum arabic as edible coating film for extending the shelf life and postharvest quality of tomato. Starch edible coatings derived from Colombian native potatoes were applied on Andean blueberry (a wild fruit native to South America) resulting in reduced respiration rate of ~27% [24]. However, previous works recommended further research focused on the improvement of the physical strength of biodegradable films comparable to that of petroleum polyfilms [22]. Sanaa and Medimagh [25] reported biomass materials that can be used to produce biodegradable and biopolymer in Africa: the vegetable cellulose extracts from cotton fibers in South Africa; *Luffa Cylindrica* in Nigeria; *Washingtonian filifera* in Algeria; *Napier* grass in Botswana; *Hibiscus sabdariffa* in Kenya, Ethiopia, and Uganda. The biopolymers such as chitosan, cellulose, and pectin were given attention in the manufacturing sector of food packaging and the research community [25]. Moreover, in Ethiopia, the film produced from pectin and chitosan extract and tested on tomato resulted in extended shelf life (15-17 days) compared to the control (10 days). Furthermore, in Nigeria, reports show that there is an intensive production of biodegradable plastic film from blending cassava starch and biodegradable polymer materials. Postharvest loss of fresh tomato on the market was reported to be 9.50, 9.80, and 10.04% in Eastern, central, and southern African countries of sub-Saharan countries, respectively [13], with Kenya, South Africa, and Nigeria recording 10.10, 10.20, and 13.40% postharvest losses, respectively [13]. Nevertheless, reduced postharvest losses among commercial or emerging farmers of tomato were achieved with the use of recyclable cardboard boxes of various sizes, bulk bins, plastic crates, and wooden crates for packaging and transportation in the South African supply chain [26].

Food packaging films can be produced either by lamination, casting, coextrusion, or coating processes from the raw plastic polymer, biopolymer, and biodegradable materials [10] (Table 1). The food packaging film is extracted from biopolymers, including gelatin, starch, cellulose, and bio-derived monomers such as polylactic acid [27]. The bacteria-derived compounds include cellulose, xanthan, curlan, and pullulan [19]. Chitosan is a natural polymer, nontoxic, edible, and biodegradable derived by deacetylation of chitin which is the second most abundant biopolymer in nature after cellulose [28]. Supplementation of different kinds of additives is recommended to improve the properties of the biodegradable film [27]. The edible biodegradable films can be stabilized by material components of hydrophilic nature such as proteins or polysaccharides. The production of films or coatings involves casting film-forming aqueous dispersions and subsequent drying. The essential oils (additives) are added to film during dispersion phase, and the mixture is achieved by homogenization or emulsification processes [21]. Thus, the dried polymer can be a structural matrix of the film and lipid droplets [21] including hydrocolloids such as edible fats, fatty acids, proteins, and polysaccharides [29]. Ivankovic et al. [19] reviewed that there are three generation stages of biodegradable polymers from which biodegradable food packaging materials can be manufactured. Accordingly, the first generation is low-density polyethylene (LDPE) film consisting of 5-15% starch filters and autoxidative additives. The second-generation films are composed of 40-70% pregelatinized starch, low-density polyethylene (LDPE), and hydrophilic copolymer additives. The third-generation materials are produced from biomaterials and can be classified into (a) polymer extracted from biomass such as starch, chitin, chitosan, plant proteins, and soybeans; (b) polymers synthesized from bio-derived monomers including polylactate and other polymers; and (c) biomonomers and polymers produced from natural or genetically modified organisms. The nanocomposite materials were identified to possess superior characteristics such as high performance, lightweight, and environmentally friendly compared to plastic food packaging materials [30]. The low cost, renewability, and availability of biopolymer are some of the desirable considerations applicable for thermoplastic starch-based food packaging materials [31].

The fruits coated with gum arabic soybean gum, jojoba wax, and glycerol resulted in delayed in changes of weight loss, firmness, and titratable acidity including delayed softening of tomato [23, 32]. The tomato fruits coated with 10-15% gum arabic film yielded less weight loss during storage period than the control sample [23]. This suggests that gum arabic film exhibited effective semipermeable barrier against O_2_, CO_2_, moisture, and solute movement, which probably decreased respiration, water loss, and oxidation reaction rates. de Jesús Salas-Méndez et al. [33] reported that the mixture of edible coatings (whey protein, glycerol, and candelilla wax) and *Fluorensia cernua* extract coated on tomato inhibited ~40% growth of pathogenic fungi. The mixture of 0.75% chitosan and 2 mM cinnamic acid coated on tomatoes yielded high firmness after 12 days of storage [34]. The film made from chitosan colloids and grapefruit seed extract (0.5-1%) inactivated *Salmonella* on cherry tomatoes during storage [35].

### 3.1. Preservation Mechanism of Edible Coatings

Quality deterioration of fruits is in function of biochemical processes in the cell structure, cell wall composition, and intracellular materials. Cellulase and polygalacturonase are two major cell wall hydrolase enzymes and were shown to correlate with softening and ripening of fruits [36]. Edible coating of fruits can delay ripening by lowering permeability of O_2_ resulting in increased intracellular CO_2_. High levels of CO_2_ can limit the activities of cell wall hydrolase enzymes and allow retention of the firmness during storage [23]. This effect of a low-oxygen environment is readily used for optimizing storage conditions and transport and for prolonging the shelf life of several fruit commodities [37]. Decreasing respiration rates of coated tomatoes could be responsible for delayed ripening and can result in reduced changes in physiological weight loss, color, titratable acidity, and retention of firmness [23]. The antimicrobial properties of edible coatings [38] can protect the fruit against firmness-degradative agents such insects and mites [39] which are carriers of fungal and bacterial spores [40] and can cause spoilage and softening of ripe tomato fruits [41]. The biodegradable packaging materials applied on fruits including tomato are decomposable and can be degraded by microorganisms in the soil [6, 42, 43].

### 3.2. Biodegradation of Biodegradable Films

Soil microorganisms can degrade biodegradable materials into natural compounds such as water, carbon dioxide, and methane including monomers such as amine, alcohol, and carboxylate acid (Table 2). Biodegradability is in function of chemical composition, nature of bonding, and water availability. The appearance of IR spectra peaks for carbonyl signals is indicative of enzymatic degradation of starch into maltose (disaccharide) and glucose (monosaccharide) [44]. The microbial action is enzymatic nature. The microbial cells exhibit saprophytic growth utilizing plant-derived metabolites as substrates [45]. The microorganisms secrete an array of amylases and cellulases responsible for enzymatic hydrolytic and oxidative breakage of glycosidic bonds in starch and cellulose. The extracellular enzymes such as esterase, cutinase, and lipase hydrolyze labile aliphatic ester linkages of plasticizing films [46]. These enzymatic processes generate metabolites that are absorbed by microorganisms for energy requirements. This is evident in the decrease and disappearance of IR spectra carbonyl signals with time. Tai et al. [44] showed significant peaks of carbonyls in 30 days and decrease after day 45, which suggested starch/cellulose breakdown and metabolite absorption, respectively. Enzymatic depolymerization of chitosan showed a sharp increase of sugars with time during 15 h and slower in 15-24 h [47]. The slower decrease of metabolites is indicative of the saprophytic phase. UV light irradiation with wavelength < 350 nm can cause chain scission of polymer molecules and can also accelerate enzymatic activity. Combined treatment of UV irradiation and cellulase enzyme degraded 60% of cellulose acetate compared to UV treatment (23%) in 7 weeks [48]. Biodegradation process is commonly characterized using thermalgravimetric analysis (TGA) reflected in three-stage degradation profiles; the first degradation corresponds to loss of water and volatiles, the second stage relates to the formation of starch subunits of lower molecular weight, and the third stage is associated with breakdown of starch components [46, 49]. The degradation of biodegradation film is in function of microbial activity in soil and water, hydrophilic nature of plasticizer, surface area of the sample, crystallinity, molecular weight of the sample, and temperature. The addition of plasticizers increases the number of polar groups and water permeability in the samples and accelerates the interaction of polar groups with water [50]. Plasticizers of biosurfactant nature possess excellent surface/interface activity and biocompatibility [51] and enhanced soil hydrocarbon biodegradation by lowering interfacial tension between soil and water [52]. Increased yields of metabolites such as volatile fatty acids were shown at optimal pH 10 under controlled fermentation process [51]. Higher pH levels can inhibit acidophilic bacteria and subsequently limiting the production of metabolites. The pulsed electric fields treated zein-chitosan-poly(vinyl alcohol) film had enhanced stability of films against electrolyte and enzyme degradation [53].

### 3.3. Properties of Biodegradable Films

#### 3.3.1. Structural Properties

The chemical structures and composition of packaging materials can be examined using Fourier transform infrared (FT-IR) spectroscopy and atomic force microscopy (AFM) [72]. Diffraction method using X-ray diffraction has been applied in the assessment and quantification of amorphous and crystalline structures in starch. The crystallinity is strongly associated with amylopectin molecule. Amylose is largely found in the amorphous lamellae, and amylopectin forms crystalline lamellae of the starch granule [73]. Crystallinity influences dispersion characteristics such as swelling of starch in plasticizers [73]. The IR spectrum is commonly characterized by the interaction of chemical bonding with IR radiations. IR spectrum for starch films exhibited broad band due to vibrational stretching of hydroxyl (-OH) groups linked inter- and intrachain. The narrow bands were associated with stretching of C-H bonds while the peaks related to carbonyl (C=O) groups attached to the ring of glucose [74]. The surface microscopic analyses of film structure were examined using scanning electron microscopy and transmission electron microscopy [75, 76]. Starch and PVA films exhibited homogenous and smooth surfaces. The cross-section of the films was the characteristic of heterogeneous and irregular (bubble like) structures which varied with degree of crystallinity. The film blends (PVA/starch) are characteristic of microstructure phase separation due to inadequate miscibility, differences in crystallinity, and extrusion method. Compatibilizer compounds such as formaldehyde and poly(ethylene glycol) are blended with films to prevent phase separation blended films [77]. Factors influencing phase separation include proportional of starch and phosphate groups in the amylopectin chain. Potato starch film did not exhibit phase separation owing to the presence of higher content of phosphate groups than other native starches. The thickness of the films determined using SEM was reported, and film blends showed higher thickness than pure starch. The differences in thickness were due to variation in molecular weight. Higher molecular weight yielded higher thickness [78]. Biodegradable edible packaging material (coating or film) has a recommended thickness of less than 254 *μ*m [9].

#### 3.3.2. Permeability Properties

The polymer matrix must exhibit effective permeability of gases for increased shelf life of food products [29]. The shelf life and freshness of vegetables and fruits including tomato are directly related to the transfer of water between the produce and the surrounding atmosphere. Thus, the primary role of packaging is to reduce the transfer of water. The poor moisture barriers in edible films were due to the hydrophilic nature of polysaccharides [29]. Lipids are hydrophobic in nature, and their inclusion in chitosan and polysaccharide films contributes to improved water vapor barrier properties. The entanglement of hydrogen bonding between NH_2_ group of chitosan and OH group of plasticizers (e.g., CAP and PVA) increased hydrophobicity of blended films (CAP/chitosan and PVA/chitosan) resulting in six reductions in water transfer rate [79]. Furthermore, Yu et al. [79] demonstrated that the addition of silica nanoparticles into biodegradable films decreased permeability of moisture. Depending on the respiratory requirements of the product and polar molecular of packaging material ingredients, oxygen permeability properties can be altered by incorporating PVC, chitosan, and silica. Oxygen permeability values were reduced by ~26% when silica was incorporated into PVA/chitosan biodegradable films [79]. The equilibrium-modified atmosphere packaging (EMAP) finds intensive application in the packaging of fresh fruit and vegetable including tomato. The EMA packaging optimizes gas transport properties according to the respiratory requirements of fresh produce. The equilibrium atmosphere is attained when the exchange of gases through the film is in steady state with the production or consumption of gases due to the respiration and transpiration processes of the fresh produce [72]. The gas transport properties can be adjusted by perforation through macroperforation and microperforation using mechanical and laser procedures, respectively [72]. Among the alternative biopolymers, starch and polylactic acid (PLA) are the major materials of interest in the research community.

#### 3.3.3. Mechanical Properties

Zhou et al. [80] developed biodegradable polylactic films using pea starch and polylactic acid for cherry tomato packaging film. However, biodegradable polylactic film exhibits poor mechanical properties compared to petroleum polylactic films [80, 81]. The biopolymers such as starch are associated with brittle films. The incorporated hydrophilic plasticizers such as polyols (glycerol, sorbitol, and polyethylene glycol) into film-forming dispersions decreased intermolecular forces and increased mobility of polymers resulting in increased flexibility and extensibility [82]. The mechanical properties (compression test, tensile strength, and strain) including film-forming capacity of the film are associated with polymer crystallinity and amylose content [82], molecular weight properties, and their distribution and concentration of additives. Plasticizing agents such as polyvinyl alcohol (PVA) and cellulose acetate phthalate (CAP) can change the mechanical behavior owing to the formation of inter- and intramolecular hydrogen bonds. The blend of starch and PVA yielded biodegradable film with better mechanical performance [83]. The film blend of chitosan-CAP and nanoZnO recorded higher tensile strength than pure chitosan film [84]. The increase in tensile strength in film blends is indicative of better interaction among the components of the film. The tensile strengths of the films increased with increasing diblock copolymer [85]. Inclusion of plasticizers and nanoparticles into starch films increased and decreased elongation at break, respectively (Table 3). Nanofillers provide reinforcement and increase interfacial bonding interaction in the film matrix. Lower molecular weight yielded higher tensile strength and elongation at break of starch films. The decrease in brittleness can be achieved by blending PLA with plasticizers such as polycaprolactone (PCL) [86]. However, the PLA-PCL blends exhibited poor gas barrier properties but can be improved using suitable fillers such as highly dispersed nanoparticles [86]. There is a need for increased research objectives to improve the mechanical properties of the biodegradable polylactic film using nanoparticles in response to the respiratory requirements of tomato fruits. Rhim et al. [87] reported that drawbacks in biodegradable polylactic film limit their full utilization in the food industry. Some of the limitations are thermal instability, low heat sealability, brittleness, low-melting length, and high water vapor and oxygen permeability [87]. Moreover, the hydrophilic nature of some biodegradable biopolymers was characterized with low water vapor barrier and consequently exhibiting weak mechanical properties [88, 89].

#### 3.3.4. Solubility Properties

The solubility values of biodegradable films are in function of hydrophilic nature of polymers. The solubility of starch film (0.208 g _dissolved_/g _dry film_) and PVA (0.19 g _dissolved_/g _dry film_) decreased in the film blend of PVA/starch (0.11 g _dissolved_/g _dry film_) [90]. This suggested a decrease in the hydrophilicity of the film matrix. The entanglement of hydrogen and hydroxyl bonding between polymers can lead to structural reorientation, thus exposing the hydrophobic nature of the film matrix and subsequently, decreasing water affinity. Nevertheless, Pellá et al. [91] reported higher water affinity of films in blended potato starch/PVA than those of pure films (Table 3). This was ascribed to an increase in -OH groups. Sajjan et al. [92] reported that lower solubility values are indicative of films with good stability in aqueous medium and are recommended for packaging applications especially for storage.

#### 3.3.5. Optical Properties

The color parameters (*L*^∗^, *a*^∗^, and *b*^∗^) and color difference (Δ*E*) are commonly measured using CIE system [93] while transmission of light and transparency [94] can be measured using UV Vis Spectrophotometer [93]. Prolonged exposure to UV and visible radiations can discolor and deteriorate the packaged food products. In view of this, transparency and UV-screening ability of packaging films are vital parameters in quality control. Generally, synthetic plastic films (low-density polyethylene and polypropylene) were reported to have lower screening ability against UV radiation [84]. The blended films loaded with nanoparticles exhibited higher absorption peaks (wavelength) than pure films. The higher surface area of nanoparticles increased the UV absorption capacity of the polymer matrix [75]. The nanocomposites (ZnO and nanoclay) increased the opacity of starch films, suggesting that nanoparticles are UV blockers and thus minimize the penetration of light.

## 4. Advances in Packaging Technology

The packaging technologies for food applications include active, intelligent, smart, modified packaging, controlled packaging, and biodegradable coatings.

### 4.1. Active Packaging

Inclusion of antimicrobial components is an aspect of innovative food packaging technologies such as active and intelligent packaging [96–101]. Active packaging is material components with the capacity to protect the packaged food from microbial proliferation [102] and provide information about the quality during transport and storage. The petroleum-based polymeric materials are commonly applied in active packaging [103]. However, environmental and safety concerns have driven research and development in packaging towards bioactive materials [103]. Active materials are intentionally added to packaging material or packaging headspace to prolong shelf life through a controlled release of antimicrobial compounds [104]. Active food packaging was developed to respond to the food market demand for improved quality of fresh produce and maintaining safety [96]. Tomato fruits preserved using active packaging resulted in extended shelf life [96], improved safety, and maintained sensory properties [97, 98]. Essential oils with antimicrobial and antioxidants activity are incorporated into food packaging films to produce active packaging materials and thus contributing to the preservation of the food [105]. Essential oils inhibit the growth of microorganisms [105]. Moreover, Azmai et al. [106] reported that coating with chitosan and cinnamic acid improved the quality attributes such as firmness and total soluble solids, reduced physiological weight loss of tomato, and prolonged the shelf life. However, global migration of compounds from packaging material into food is a food safety concern and can cause contamination [107]. Bradley et al. [108] postulated that intelligent food packaging can cause toxicological risk, environmental contamination, and problems with recovery and recycling of the packaging materials. The package of active biodegradable corrugated cardboard tray tested on cherry tomato was reported to extend the shelf life of tomato for a month [96].

### 4.2. Active Scavenging and Adsorbents

The liquid exudate from fresh tomatoes influences sensorial and microbial quality [109]. The adsorbent pads are designed to take up the exudate and ultimately preserving integrity and quality of packaged products [110]. The active scavenging systems remove gases such as CO_2_, O_2_, and ethylene from the package or container. The presence of oxygen in package accelerates oxidation or spoilage. The decreased reactive oxygen species was associated with delayed overripening and decreased susceptibility to *Botrytis cinerea* [111]. The role of scavenging was achieved using flavonoids produced from different tomato varieties [111]. Ethylene scavengers (KMnO_4_, activated carbon, clay, and zeolites) have been applied on fruits and vegetables including tomatoes. The KMnO_4_ transforms ethylene into acetate and ethanol. Cherry tomato treated with 0.1% (v/v) ethanol during storage resulted in elevated ascorbic acid, sucrose, and fructose contents, inhibited ripening, and improved sensorial quality [112]. The KMnO_4_-based technology has been reported to have a limited commercial application due to uncertainties on its effectiveness as postharvest tool and also concerns relating to health, environmental, and safety [113]. However, KMnO_4_-promoted nano zeolite was reported to show high ethylene removal efficiency [114]. The condensation due to transpiring tomatoes can lead to accumulation of moisture. The removal of moisture can be achieved using active element (silica gel, polyacrylate salts, zeolites, and microporous clays) in the packaging system [115]. A sodium polyacrylate-cotton mixture applied as moisture adsorbent in the form of sachets resulted in noncondensation of water in active packaging system of tomato fruits [115]. The preservative releasers based on the blend of itaconic acid and chitosan enriched with tomato bioactive extract yielded significant antimicrobial effects on packaging films [116]. Other preservative releasers applied in packaging system for tomato include silver zeolite, organic acids, spice/herb extract, vitamins C and E, sorbates, chlorine dioxide/sulfur dioxide, and benzoates and propionates [117].

### 4.3. Intelligent and Smart Packaging for Tomatoes

Intelligent packaging is a packaging that comprises of external or internal indicators that give information about the history on safety and quality of the product [104]. Vanderroost et al. [118] reviewed that smart or intelligent packaging technologies offer the opportunity to record and detect changes in the packaged product and its environment [118]. Intelligent packaging tracks the history of the food along the supply chain [97]. For instance, Bartkowiak et al. [119] reported that the lactic acid-based time-temperature indicators [102] provided history on quality and time-temperature of lactic acid-based food. Hence, this application can find use in tomato and tomato-derived products that are acidic in nature. However, a few of such technologies were commercialized, partly due to higher cost of investment. Lee et al. [97] suggested low-cost intelligent packaging material production for food industries.

## 5. The Fourth Industrial Revolution in Packaging and Tomato Supply Chain

The major technological drivers for the fourth industrial framework (4IR) are physical, digital, and biological technologies [120]. The appropriate technology driver for packaging and tomato supply chain is digital technology which includes fields such as artificial intelligence and robotics, linked sensors (Internet of Things), virtual and augmented realities, additive manufacturing (3D bioprinting organic tissues), advanced materials, and nanomaterials [121]. In agricultural production, the digital technology finds application in areas of smart sensing and monitoring, smart control, smart analysis, and planning [122]. The notable digital technology in packaging and tomato supply chain is the use of sensors and electronic nose for classification and discrimination of germplasm of food crops, quality control, and verification and authentification of geographical origin. Traditionally, wet extraction and analysis is a common laboratory approach of obtaining key trait information about tomato germplasm in different agroecological zones; however, this approach involves the use of chemicals which are detrimental to the environment and human safety. Levin [121] outlined methodological approach required to achieve smart sensing digital systems in the quality analysis of food crops: (i) sample handling systems, (ii) detection systems, and (iii) data processing systems (Table 4).

### 5.1. Sample Handling System

The conventional isolation techniques for volatile compounds such as steam distillation and solvent extraction can cause modification to quantity and quality of flavor profiles in samples [123]. In addition, these techniques are destructive and time-consuming. The rapid techniques include the purge and trap headspace sampling method [123, 124]. The headspace can be in static or dynamic mode. This method involves trapping and concentrating volatile compounds on a solid support which is then heated to release volatiles into gas chromatography (GC) or GC/mass spectrometer (MS) systems containing sensing elements. The purge and trap and dynamic headspace sampling were used to extract the flavor compounds from tomato fruits [125, 126]. The static headspace sampling methods in tomatoes [127] extracted a true reflection of flavor profile but yielded low amounts of compounds, suggesting loss of volatiles during sample handling and may result in undetection. Such shortcomings were eliminated with the use of cold trapping static headspace. This cryofocusing technique allows samples to be concentrated without heating. The solid-phase microextraction (SPME) is a user-friendly preconcentration method. In this technique, volatile components interact and react with fiber-coated probe inserted into the headspace of a sample and then transferred to a GC injection port where the volatiles are desorbed. The SPME has been applied in the analysis and discrimination of volatiles in tomato landraces [128]. The stir bar sorptive extraction is another sampling technique in which a magnetic bar coated with polymers is suspended in the headspace. This technique is similar to inside-needle dynamic extraction method, a preconcentration technique in which absorbing polymers are fixed inside the needle, and enables the interaction of polymers with volatiles [129]. The mechanism of volatile release, different types, and factors guiding the selection of stir bar sorptive were reported [129].

### 5.2. Detection System

The detection system is the application of an array of sensors operating as devices to identify chemical compounds in the headspace. Chemical sensor transforms chemical quantity into an electrical signal in function of the concentration of specific atoms, molecules, or ions in gaseous or liquid forms [124, 130]. The sensors applied in e-nose are capable of responding to molecules or particles which are volatile in nature and can vary with relative molar masses. Several sensor arrays used in the development of e-nose have been reported. Piezoelectric sensor is a device that utilizes acoustic waves generated by piezoelectric materials such as quartz or LiNbO_3_ [130] to detect changes in pressure, acceleration, temperature, strain, or force and converting them to an electrical charge [131]. The acoustic (piezoelectric) impulse response parameters (dominant frequency, firmness index, and elasticity coefficient) yielded a good to strong correlation with firmness parameters (compression force and puncture force) of tomatoes during storage time [131]. Electrochemical sensors are devices that convert electrochemical reactions between an electrode and analyte into an output signal specifically related to the concentration or partial pressure of the gaseous species [132]. The types of electrochemical sensors include potentiometric, conductometric, amperometric, and voltametric but they have limited detection limits [130, 132–134]. The recent areas of research in electrochemistry involve the modification of electrochemical sensors using conductive materials such as nanoparticles to enhance their response and detection limits [135]. The sensitivity of a conductive material-based sensor is defined by change in the electrical conductivity of the semiconducting material when exposed to test volatiles. Nanoparticles such as spherical Cd_2_SnO_4_ and Zn_2_SnO_4_ provide large surface area for the absorption and have high electron density [136]. The electrochemical DNA sensor was developed to perform the direct determination in intact genomic DNA extracted from tomato seeds [137]. This suggests that electrochemical sensor can be used to discriminate the bionature such as organic or inorganic germplasm in tomato cultivars. Other detection systems include and optical and thermal sensors. The optical sensors which include absorbance, reflectance, luminescence, and surface plasmon resonance techniques [138] are nondestructive methods based on multispectral three-dimensional (3D) imaging [139]. The fertilizer application and irrigation water were optimized based on the reflectance characteristics of the canopy such as leaf temperature, leaf relative water content, and leaf chlorophyll content in the field of tomato [138]. Thermal sensors detect heat produced by a specific analyte in the chemical reaction. The different types of heat sensors include resistance temperature detectors (RTDs), thermocouples, thermistors, infrared sensor, and semiconductor sensors. Thermal sensing depends on analyte change of state in response to temperature and light. The signals of optothermal window/light-emitting diode correlated strongly with color-related quality parameters of tomato-derived products [140]. The problem associated with e-nose is that they tend to produce limited information by targeting specific measurements. In real time, the food ground matrix (fresh or processed) is a complex of interacting volatile constituents. Peris and Escuder-Gilabert [130] proposed a sensor hybrid system to generate different sensor outputs in a single spectrum. Nevertheless, this would require the application of more complex electronics combined with standardized sensor outputs. The problem associated with e-nose such as masking of sample constituents, influence of moisture, and nonlinearity of signals were solved by integrating e-nose system with mass spectrometry. The MS-e-nose integrated systems are a new technology that introduces volatile compounds into the ionization chamber of MS-based instrument that produces an output of ion-fragmentation patterns [130] representing a chemical footprint for volatile compounds in a sample. The MS-based e-noses find application in qualitative analyses of alcoholic beverages.

### 5.3. Data Processing System

The sensor array output of samples is processed using pattern recognition techniques [141]. The interaction of volatile compounds with sensing elements produces changes in the electrical resistance of the sensor. The changes in electric signals are different depending on the sensor kinetics and thus a variety of signals collected and remitted into data acquisition and processing unit in which a volatile fingerprint can be interpreted using appropriate mathematical recognition techniques such as principal component analysis (PCA), linear discriminant analysis (LDA), and artificial neutral network (ANN). The discrimination of geographical origin and identification of different olive oil varieties were achieved based on metal oxide semiconductor sensor using PCA and LDA and yielded ~98% and 96% recognition success rates, respectively [141]. Optimization of recognition pattern requires the use of an array of sensors commonly in the range from 1 to 32 sensors. The sensors are evaluated using a loading analysis of PCA to identify significant patterns and their corresponding sensors. PEN2 with 10 different metal oxide sensors (MOS) was used to recognize the ripening state of tomato [142], and PCA biplot loadings showed that sensor MOS 2, 6, and 8 were located on the extreme positive coordinates of the biplot but MOS 2 had variance of ~95% (please see figures in Gómez et al. [142]). This indicated that the three sensors were extremely distinguished; however, MOS 2 exerted higher influence on the ripening pattern of tomato. In addition, sensors 6 and 8 clustered together, which is an indicative of similarities in their response to ripening.

### 5.4. Applications of Virtual Platforms in Traceability

The conductivity of sensors is in function with changes in physicochemical characteristics of the product. The sensor PEN2 e-nose (Airsense Analytics, GmBH, Schwerin, Germany) was used to detect quality changes (soluble solids content, pH, firmness, and vitamin C) in juice extracted from cherry tomato [143]. In the same study, Hong and Wang [143] analyzed the sensorial characteristics of tomato juice using *α*-Astree e-tongue (Alpha MOS Company, Toulouse, France). Berna et al. [144] compared two electronic nose systems, quartz microbalance-based electronic nose (E-nose) and a mass spectrometry-based electronic nose (MSE-nose) against gas chromatography (GC) as a standard reference in the analysis of aroma differences among tomato cultivars. The MSE-nose produced variation while E-nose hardly discriminated the differences among the cultivars [144]. The electronic sensories (e-nose and e-tongue) are commercial ready on the markets; however, their application would require validation studies specific to genetic factors, geographic locations, growth traits, and changes in postharvest handling and logistics. Other studies reported analysis of sourness, saltiness, and umami using electronic nose and electronic tongue coupled with gas chromatography-mass spectrometry (SPME/GC–MS) [145].

The unpredictable changes in supply chain, dynamics in quality, and regulatory system requirements for food safety and sustainability would require networked processes of virtualization to enable centralized operational management of food supply chains [146]. This is aimed at achieving a food supply chain that can be monitored, controlled, planned, and optimized in real-time using the Internet-based virtual objects instead of on-site physical observation [146]. The RFID (radio frequency identification), EPC global (Electronic Product Code), and ebXML (Electronic business using eXtensible Markup language) are some of the electromagnetic or electrostatic coupling technologies commonly applied to virtualization of supply chain traceability for commercial products including food and petroleum-based plastic materials. The European Union regulatory requirements for traceability of food contact materials are mandatory [147]. South Africa is among the major regional tomato producer in sub-Saharan Africa and ranks among the major exporters of fresh produce including tomato to the EU [148, 149]. However, no information relates to the traceability of biodegradable packaging materials in the South African tomato supply chain. The authentification of biodegradable packaging can be assured by developing a footprint characteristic of a material component to enable digital differentiation between biodegradable and synthetic plastic packaging materials. The biodegradable packaging materials are considered suitable for organically produced agricultural products including tomato. Literature showed that there are several benefits of supply chain traceability including enhanced integrity of a supply chain, easy tracking of product from farm to consumer, tracing of products to their origin, avoiding the risk of inappropriate labeling of products, and improves effectiveness of product audits.

**Figure 1:**
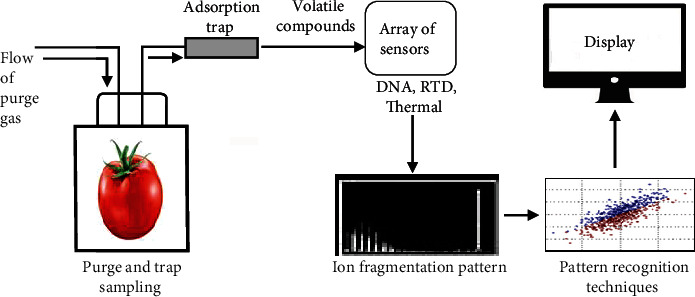
Traceability of tomato fruits in function of environmental conditions including soil fertility, geographical source, type of germplasm, type of coating, and postharvest distribution and storage conditions.

There are concerns with packaging labeling regarding the misrepresentation of package, package ingredients, and false statements aimed at making an economic gain. This desire to gain a profit by mislabeling of products is a concern in the markets. Consumers are increasingly becoming aware of the value of food quality and safety. The contaminants resulting from nonbiodegradable packaging materials represent an important food safety topic [150, 151] and can lead to decreased consumer confidence in finished/processed food products. Subsequently, such safety concerns have stimulated interest in authentification and traceability for compliance with the regulations, consumer protection, and competition. The packaging products must reflect the origin of the material ingredients, details of postharvest treatments, and the geographic location (Figure 1). Turci et al. [152] reported that the internal traceability has been established as the reliable approach of preventing fraudulent or deceptive labeling and also to certify originality and quality of tomato products on the market and their postharvest influencing factors including packaging material nature. The commonly documented parameters for authentification and internal traceability for tomatoes are protein [153], metabolite [154], and DNA [152]. There is a need to identify nanoparticle makers for traceability and authentification of biodegradable materials.

The nanosensor signals expressed in nanometers are developed to detect changes in structural and functional properties of materials at nano level (1nm = 10^−9^) [155] and are embedded in food packaging material to monitor freshness of perishable products [156] during production, processing, and distribution. The suitability of nanomaterials is in function of good mechanical and electrical properties [157] and high surface area [155]. The tracking of food ingredients using nanosensor through the processing chain [158] suggests the potential application of nanodevices to monitor the ingredients of biodegradable materials. The structural differences between natural biopolymers and synthetic polymers [159] can be streamlined at nanoscale to develop differentiating markers. The data on structural and functional properties of material components in response to electromagnetic behavior can lead to the development of nanodevices to enable the identification of material ingredients and formulations. The biodegradable materials can be degraded by enzymatic action of living organisms (bacteria, yeasts, and fungi) and storage conditions (humidity and water). There is a gap in research for mathematical modeling relating to impact of degradability agents on the durability and mechanical integrity of biodegradable materials. The network of nanosensors can be implemented to achieve product monitoring and environmental conditions. The RFID and wireless sensor network (WSN) integration was suggested [160] to capture environmental information along with product tagging and thus assuring the end-user on meeting the system requirements throughout product delivery and storage such as maintaining the required temperature and humidity [160]. The loss of sensor data occurs due to corrupted network or hardware failure [160, 161]. The missing data can be predicted by data mining techniques [160] using interpolation methods: k-nearest neighbors (KNN) [161], global refinement method Delaunay Triangulation, PCA, multichannel singular spectrum analysis (MSSA), and compressive sensing [161]. The RFID-WSN can be integrated with data mining techniques to incorporate the data due to changes in the storage conditions. The novel environmental space-time improved compressive sensing (ESTI-CS) algorithm [161] achieved environmental reconstruction with a minimal error of 20% for 90% corrupted network. However, there is limited information on the implementation of nanosensor technology in integrated sensor system.

## 6. Conclusions and Recommendations

The demand to replace synthetic plastic with biodegradable packaging materials is increasing. The development of biodegradable packaging is influenced by several factors including policy and legislative changes and world demand for food and energy resources. The biodegradable materials are associated with poor properties (high brittleness and low transparency). Nevertheless, the use of nanocomposite ingredients can improve brittleness and other physical properties. There are limited studies focused on interactions between the polymers and the food products. In addition, there are few studies that point to toxicities associated with the global migration of ingredients from a biodegradable package into the food. The appreciable use of digital platforms in the tomato industry to attain objectives of 4IR would require great amount of data to develop a hybrid sensor response in function of production (agronomy and genetic traits), postharvest treatment, storage conditions (temperature and relative humidity), quality traits, and geographical origin of genetic factors. There is a need to develop fingerprint markers to enable differentiation and authentication of biodegradable materials.

## Figures and Tables

**Table 1:** Biodegradable packaging films and applications.

Biodegradable film	Substrate	Production	Suitability for application	Reference
Polylactic acid (PLA)	Sugars or impure carbon substrates (starch, molasses, or whey)	Two-stage degradation processes: (1) hydrolytic and (2) enzymatic	Compositing and lamination	Nilsuwan et al. [54]
Corn starch/blueberry	Corn starch, blueberries (*Vaccinium corymbosum* L.)	Starch extraction and production of blueberry powder	pH indicator (rich in anthocyanin, changes color in different pH conditions)	Luchese et al. [55]
Starch/PLA canna	Mixture of PLA, compatibilizer, starch, and zinc stearate	Mixing (miscibility)	Thermal stability of antimicrobial activity	Mania et al. [56]; Morales and Calle [57]
Cellulose nanofiber	Fruit fiber	Steam pressure and water treatment and neutralization and drying; wet grinding	High heat resistance, good discharge capacity, and improved electrolyte wettability	Sun et al. [58]
Chitosan	Shells of shrimps (chitin)	Washing shells, dried, and mesh homogenizing; demineralization, deproteinization, and deacetylation	Coating, biocompatibility, anticholesteremic, ion sequestering actions, and antimicrobial activity	De Queiroz Antonino et al. [59]
Chitosan/cassava starch	Cassava starch, ~90% DD chitosan	Two-stage processes: starch casting and coating	Coating	Bangyekan et al. [60]
Chitosan/PVA/PCL	83% DD chitosan, PVA, and PCL	Dispersion processes, heating, and mixing	Laminating and coating	Yar et al. [61]
Chitosan/PVA	~85% DD chitosan	Dispersion of chitosan and mixing; crosslinking of mixture at freeze-thaw cycles		Bi et al. [62]
Chitosan-fungal	75-85% DD chitosan, mushroom (*Tricholoma terreum*)	Mixing chitosan and mushroom extract; film-forming by casting.	Antimicrobial and antioxidant	Koc et al. [63]
Protein film	Dehydrated lentil	Protein extraction and purification, mixing, and thin casting	Crosslinked by transglutaminase	Tinoco et al. [64]
Protein-lipid film	Soybean	Extraction of soymilk slurry, ohmic heating/water bath heating, and film casting and drying	Improving hydrophilicity	Lei et al. [65]
Ozone-starch film	Potato, ozone	Starch extraction, dispersion, ozonation, gelatinization, casting, and drying	Increases number of carbonyl and carboxyl groups	La Fuente et al. [66]
Cassava starch/evan film	Starch, *Bacillus subtilis* natto CCT 7712	Production of microbial levan	Edible film, coating, antioxidant, anti-inflammatory, anticarcinogenic, anti-AIDS, and hyperglycaemic inhibitor	Mantovan et al. [67]

DD: degree of deacetylation.

**Table 2:** Degradation methods of biodegradable material films.

Film	Biodegradable medium	Biodegradability	Reference
Starch	Aerobic biodegradation	60% disintegration rate (CO_2_ produced) in ~10 days; three-stage TGAs: first degradation ~61–63°C [68], second degradation ~257°C, and maximum disintegration *T*_p_ ~280–290°C	Tampau et al. [46]
Cassava starch/yerba mate	Decomposition: vegetal compost	Degradation time 6-12 days exhibited changes in tonality and breakdowns materials. Three-stage TGAs: first degradation ~100–150°C [68], second degradation ~180–60°C, and maximum disintegration *T*_p_ ~250-350°C	Jaramillo et al. [49]
Cassava starch/yerba mate	Acid and alkaline stability treatment	Swelling capacity: ~1.6 in acid and <1.9-2.2 in alkaline condition	Jaramillo et al. [49]
Zein-chitosan-poly(vinyl alcohol)	*In vitro* degradation (enzymatic susceptibility)	Amine content: ~0.03 mM Scrine Eq in 30 min; amino acids increase between 60 and 260 min (0.08-0.04 mM Scrine Eq)	Giteru et al. [53]
Zein-chitosan-poly(vinyl alcohol)—PEF treated (between 60–70 kJ/kg and 600–620 kJ/kg)	*In vitro* degradation (enzymatic susceptibility)	Amine content: ~0.02 mM Scrine Eq in 30 min; amino acids increase between 60 and 260 min (0.02-0.04 mM Scrine Eq). Higher energy yielded higher amino acids	Giteru et al. [53]
Poly(L-lactide)	Combination of UV irradiation and enzymatic degradation	Erosion depth deepens with increasing degradation time	Kikkawa et al. [69]
PLA	Hydrolytic degradation	Increased mass loss as a function of immersion time (*t*) at pH = 10, complete degradation in 288 h; other pH = 4 and 7 yielded no changes in mass loss	Scaffaro et al. [70]
PLA/CRV	Hydrolytic degradation	Faster kinetics of hydrolytic reactions compared to PLA	Scaffaro et al. [70]
Poly(vinyl alcohol)/chitosan	Buried in the soil for 30 days	60% weight loss at 30 days	Yu et al. [71]
Poly(vinyl alcohol)/chitosan SiO_2_	Buried in the soil for 30 days	~40% weight loss at 30 days	Yu et al. [71]

TGA: thermalgravimetric analysis; CRV: carvacrol (CRV) essential oil (2-methyl-5-(1-methylethyl)-phenol); PEF: pulsed electric fields.

**Table 3:** Properties of pure and blended biodegradable packaging films.

Samples	OTR	WVTR (g/day/L)	Solubility	Tensile (MPa)	EB (%)	Thickness (*μ*m)	Reference
Potato starch			0.28	5	100	182	Gómez-Aldapa et al. [83]
PVOH			0.25	35	650	109	Gómez-Aldapa et al. [83]
Potato starch: PVOH			0.24-0.35	6-15	110-450	133-177	Gómez-Aldapa et al. [83]
Polylactic acid (PLA)	200	66		45	2.5	100	Ivonkovic et al. [95]
PLA				38	16		
PLA-CRV				24	29	57	
ChitosanMw = 300kDa		13 × 10^−11^ g m-^1^s^−1^Pa^−1^		20	20	71	Liu et al. [78]
ChitosanMw = 150kDa		14 × 10^−11^ g m-^1^s^−1^Pa^−1^		23	24	70	Liu et al. [78]
ChitosanMw = 50kDa		16 × 10^−11^ g m-^1^s^−1^Pa^−1^		25	28	69	Liu et al. [78]
Chitosan-kojic film		3 − 9 × 10^−11^ g m-^1^s^−1^Pa^−1^		25-55	29-65	90-124	Liu et al. [78]
Chitosan film		6.6		5	50		Khamhan et al. [85]
Chitosan-nano^∗^		5-6		7-12	15-25		Khamhan et al. [85]
Chitosan				82	5.2		Suyatma et al. [28]
Chitosan-PLA				52-72	3.6-4.9		Suyatma et al. [28]
Chitosan	1850	438		8	~13		Indumathi et al. [84]
Chitosan/CAP	1832	390		~9	29		Indumathi et al. [84]
Chitosan/CAP-ZnO	1490-1724	120-160		9-11	15-26		Indumathi et al. [84]

^∗^Methoxy poly(ethylene glycol)-*b*-poly(ɛ-caprolactone) diblock copolymer, cellulose acetate phthalate (CAP); O_2_ TR: oxygen transfer rate at 0% RH; WVTR: water vapor transfer rate at 100%; CRV: carvacrol (CRV) essential oil (2-methyl-5-(1-methylethyl)-phenol).

**Table 4:** Application of sensors in tomato and fruits.

Sample	Objective	Sampling	Detection	Data processing	Reference
Tomato (heat wave)	Discrimination between ripeness states	SHS	Libra nose: 5 QMBs	PCA	Peris and Escuder-Gilabert [130]
Tomato (heat wave)	Discrimination between ripeness states	SHS	PEN 2: 10 MOS	PCA, LDA, and PLS	Peris and Escuder-Gilabert [130]
Heat wave	Discriminating shelf life during two storage treatments	SHS	PEN 2: 10 MOS	PCA, LDA, and PLS	Peris and Escuder-Gilabert [130]
Tomato plants	Diagnosis of aphid-infested tomato plants	SPME	GCMS-QP2010 SE	PCA	Cui et al. [162]
Tomato seedling	Detecting damage caused by mold and blight	SHS	PEN 2: 10 MOS	PCA, LDA, and BPNN	Cheng et al. [163]
Tomato fruit	Classification of odours	SHS	EN: 6 MOS	PCA	Kasbe et al. [164]
Date pits	Assessing stability of 32 sensors	PTHS	PEN: 32 sensors	PCA	Rahman et al. [165]
Tomato	Monitoring flavors	SHS	PEN3: 10 MOS	PCA, LDA	Xu et al. [166]
Tomato	Field phenotyping of key traits (SSC, glucose, fructose, TA, citric acid, ascorbic acid, malic acid, and lycopene)	ATR surface	DTGS	PLSR	Akpolat et al. [167]
Tomato	Evaluating ripening state	SHS	PEN2: 10 MOS	PCA	Gómez et al. [142]

PLSR: partial least squares regression; ATR: attenuated total reflectance; DTGS: deuterated-triglycine sulfate detector; SHS: static headspace; MOS: metal oxide sensors.
